# Trends in Pathological Autopsy in Japan From 1958 to 2023

**DOI:** 10.1111/pin.70038

**Published:** 2025-07-16

**Authors:** Hiroshi Uozaki, Yoshinao Kikuchi, Masato Watanabe

**Affiliations:** ^1^ Department of Pathology Teikyo University School of Medicine Tokyo Japan

**Keywords:** autopsy rate, COVID‐19, Japan, life expectancy, pathological autopsy, vital statistics

## Abstract

Pathological autopsies are essential for medical education and medical progress, yet their numbers have been declining globally. *Annual of the Pathological Autopsy Cases in Japan* (APAC‐J), established in 1958, is a comprehensive nationwide database of autopsies performed in Japan. This study analyzed APAC‐J data from 1958 to 2023, encompassing over 1.49 million cases. The number of autopsies peaked at 40,680 in 1985 but declined significantly to 10,020 in 2019, and further to 6,557 in 2022 largely due to the COVID‐19 pandemic. Autopsy rates declined after the medical school conflicts in the late 1960s, with temporary increases following the introduction of board certification for pathologists. The number of data‐reporting facilities rose to 934 by 2019. Since 2000, the proportion of brain dissections has slightly increased, while limited autopsies have decreased (24% and 3.9%, respectively, in 2023). The male‐to‐female ratio is over 2.0, and average ages of autopsy cases remain over 10 years younger than Japanese life expectancy. Autopsy rates were higher among individuals from childhood to middle age. This study demonstrates that social dynamics and healthcare system reforms have influenced autopsy practices. Interpretation of autopsy case groups must consider demographic characteristics and shifts in autopsy implementation over time.

## Graphical abstract

Autopsy trends across Japan from 1958 to 2023 were analyzed using all 66 volumes of *Annual of the Pathological Autopsy Cases in Japan* (Japanese Society of Pathology). The number of annual autopsy cases peaked at 40,680 in 1985, followed by a continuous decline, which was further exacerbated by the COVID‐19 pandemic (6,557 in 2022). Conversely, the number of autopsy facilities increased, reaching 934 in 2019, and changes in the age and sex distribution of the autopsy cases were also observed.

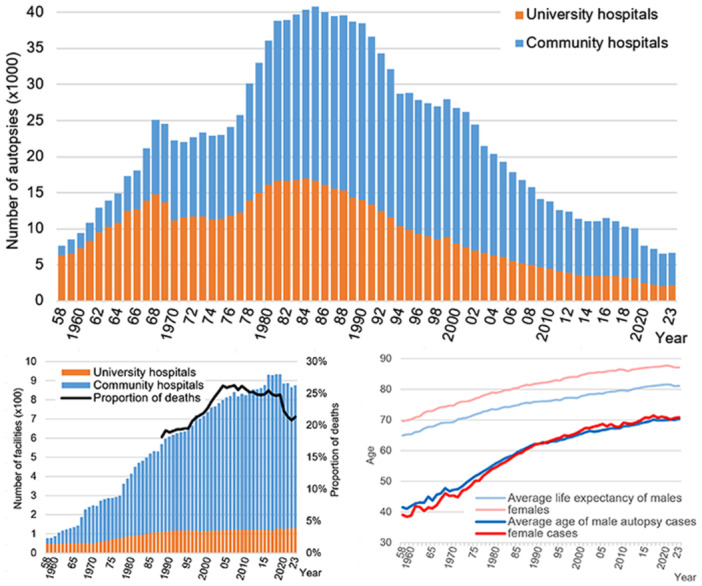

## 1 Introduction

Autopsy plays an essential role in advancing medicine and improving clinical practice [1]. Its importance was further underscored during the recent COVID‐19 pandemic, where it provided valuable insights into pathogenesis [2, 3, 4].

‘*Annual of the Pathological Autopsy Cases in Japan*’ (APAC‐J) includes pathological autopsy cases in Japanese. The inaugural volume, compiling autopsy cases from 1958, was published in 1960 by the Japanese Society of Pathology (JSP) [5, 6]. Since then, APAC‐J has been issued annually, collecting pathological autopsy cases from all across Japan. Each case entry includes age, sex, clinical diagnosis, and a succinct pathological diagnosis limited to about 100 characters. Post‐mortem biopsies, which are referred to as ‘necropsies’ in Japan, are not included in APAC‐J.

Reports on autopsy practices have also been published from other countries. However, many studies include a mix of forensic autopsies, are confined to a limited number of hospitals even when focusing exclusively on pathological autopsies, or present registry data from specific regions, making it challenging to capture a comprehensive picture of pathological autopsies. While some studies have utilized APAC‐J, none have conducted a thorough, long‐term analysis that synthesizes findings from multiple perspectives.

In this study, we retrospectively analyzed the implementation status of autopsies in Japan over the past 66 years, two‐thirds of a century, based on a detailed review of the APAC‐J.

## 2 Methods

We used all 66 volumes of APAC‐J, in which over 1.49 million cases have been recorded. Many forensic cases were mixed in APAC‐J before 1966, but they were excluded. Forensic autopsies were carefully excluded, even when they occurred in just a few hospitals. In this article, ‘autopsy’ refers to a non‐forensic, pathological autopsy.

We obtained data on mortality rates and life expectancy at birth in Japan from Vital Statistics [7]. We calculated the nationwide autopsy rate by dividing the number of autopsies by the total number of deaths nationwide.

## 3 Results

### 3.1 Autopsy Numbers and Rates

#### 3.1.1 Overall Trends

**Figure 1:**
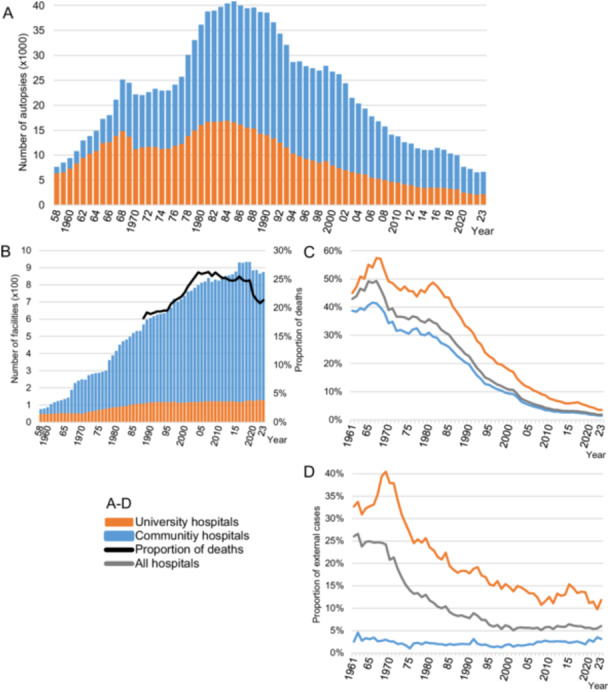
Trends in the implementation of pathological autopsies in Japan**.** (A) Trend in registered cases in APAC‐J (1958–2023). (B) Trend in the number of data‐registering facilities and the proportion of deaths in these facilities relative to national deaths (1958–2023, 1988–2023). (C) Trend in intrahospital autopsy rates (1961–2023). (D) Trend in the proportion of external cases (1961–2023).

After an initial increase in registered cases per year until 1968, there was a slight decrease, followed by a continuous increase until the 1980s, reaching a peak of 40,680 cases in 1985 (Figure 1A). There was a subsequent decline, with a further significant decrease observed during the COVID‐19 pandemic starting in 2020, with 6,557 cases in 2022. The number of registering facilities began at 76 in 1958 and had increased to 934 by 2018 (Figure 1B).

The trend in intrahospital autopsy rates at registering facilities is shown in Figure 1C. Atomic Bomb Casualty Commission (ABCC) and Radiation Effects Research Foundation (RERF) were excluded from the calculation of intrahospital autopsy rates due to their unique characteristics. The intrahospital autopsy rate peaked around 50% in 1967, declining to 1.8% by 2023. The decline in autopsy rates within hospitals began before the decrease in nationwide autopsy numbers.

The trend in proportion of external cases is shown in Figure 1D. ABCC and RERF were excluded. The proportion of external cases at university hospitals has gradually decreased from approximately 40% around 1970. The proportion of external cases at community hospitals is significantly lower than that at university hospitals, remaining relatively constant at 2%–3%, but has recently shown a slight increase (3.6% in 2022).

#### 3.1.2 The Impact of University Conflicts

**Figure 2:**
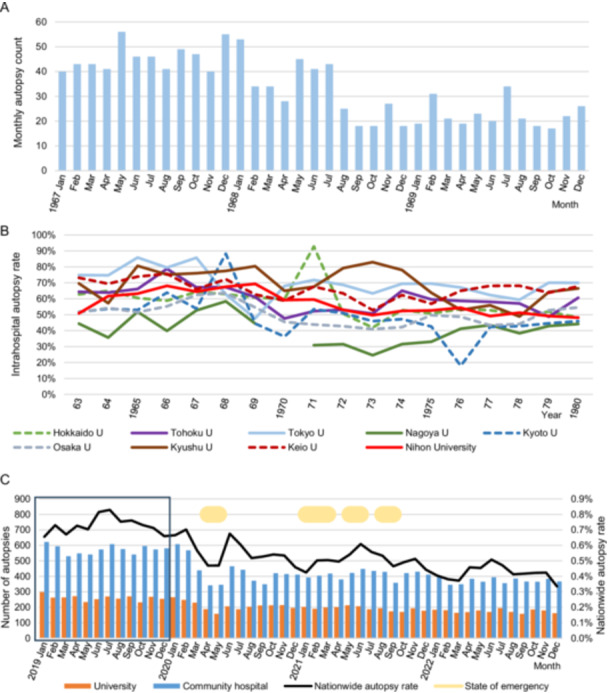
Autopsy implementation status in specific periods**.** (A) Monthly autopsy numbers in The University of Tokyo from before to after the university conflict period (1967–1969), (B) Trends in annual intrahospital autopsy rates in the seven former imperial universities and two private universities (1963–1980), (C) Trends in monthly autopsy numbers and the nationwide autopsy rate from before to after the COVID‐19 pandemic (2019–2022).

Given the potential impact of the 1968 university and medical school conflicts on the observed reduction in autopsy numbers around 1970, we investigated monthly autopsy trends at The University of Tokyo, a site notably affected by these conflicts, spanning the period before and after the unrest (Figure 2A). In 1968, the university experienced widespread disruption, including university‐wide strikes, the lockdown of the School of Medicine's main building, and the seizure of Yasuda Auditorium [8].

Because the intrahospital autopsy rate declined in the 1970s, particularly in university hospitals, we investigated the trends in individual intrahospital autopsy rates at the seven former Imperial Universities, as well as two private universities, Nihon University and Keio University, where major university disputes occurred (Figure 2B). Variations in autopsy rates were observed among the former Imperial Universities. A notable decline in the autopsy rate at The University of Tokyo was evident in 1969. Data for Nagoya University is missing for 1970 due to the unavailability of in‐hospital mortality numbers; however, a decrease in the autopsy rate was observed for approximately 5 years starting in 1971. In contrast, Kyushu University maintained a high autopsy rate throughout the early 1970s.

#### 3.1.3 The Impact of COVID‐19

To investigate the impact of the COVID‐19 pandemic, we collected monthly autopsy numbers and nationwide autopsy rates from 2019 to 2022 (Figure 2C). In Japan, emergency declarations were issued four times in response to COVID‐19, significantly affecting people's lives and economic activities. There was a significant decrease in autopsy numbers during the first emergency declaration. Even after the declarations were lifted, the number of autopsies remained low until the end of 2022.

In 2019, just before the COVID‐19 pandemic, the data within the square in Figure 2C show relatively stable monthly autopsy numbers, with a winter dip in the national autopsy rate. This trend has been consistently observed back to 1965 (Figure S1).

### 3.2 Characteristics of Cases

**Figure 3:**
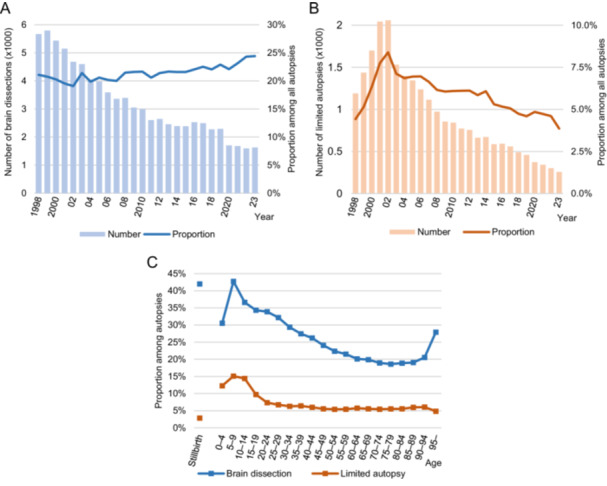
Brain dissections and limited autopsies. (A) Trend in the number of brain dissections and their proportion among all autopsies. (B) Trend in the number of limited autopsies and their proportion among all autopsies. (C) Proportion of brain dissections and limited autopsies among all autopsies by age group (1998–2023).

Since data collection began in 1998, the rate of brain dissections among autopsies has gradually increased from 20% in the early 2000s to 24% by 2023 (Figure 3A). A limited autopsy was defined as an autopsy in which a complete examination of all major truncal organs was not performed. Limited autopsies accounted for 8.4% of all autopsies in 2002, but the proportion decreased to 3.9% by 2023 (Figure 3B). The proportion of brain dissections and limited autopsies by age group is shown in Figure 3C. Brain dissections were more frequently performed in children and young adults, as well as in the very elderly. Limited autopsies tended to be more common in children, while the proportions were relatively consistent across other age groups.

**Figure 4:**
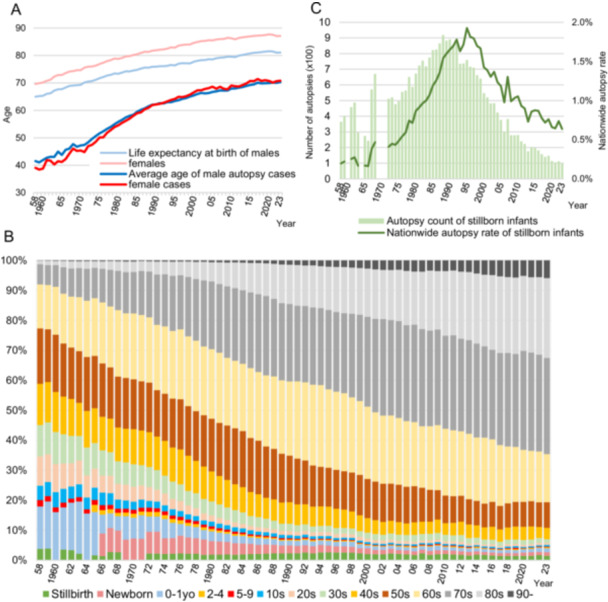
Trends in the age of autopsy cases. (A) Trends on average age of autopsy cases and life expectancy at birth in Japan. (B) Trend in age group composition. The data from 1958 to 1964 include several forensic autopsy cases, and children aged 2–4 are classified in the 0–1 age group. The period from 1958 to 1965 includes newborns in the 0–1 age group. Data for the years 1960 and 1964 do not include stillbirths. Stillbirths are included in the 0–1 age group for the years 1969–1971. (C) Trends in stillbirth autopsy numbers and nationwide autopsy rates. Some years had no data available for analysis.

The age trends of autopsy cases are shown in Figure 4A. The average ages up to 1973 were estimated based on the age distribution tables. Forensic cases from Tokyo Metropolitan Medical Examiner's Office are included up to 1964 in Figure 4. The average age has increased in parallel with the rise in life expectancy, but the increase in the average age of autopsy cases has outpaced the increase in life expectancy. While the life expectancy differs by approximately 6–7 years between male and female, the age difference in autopsy cases between sexes is minimal. Recently, the average age of autopsy cases is about 11 years lower than the life expectancy for male and about 16 years lower for female. Figure 4B illustrates the alteration in the age composition of the case series. Data for the years 1960 and 1964 do not include stillbirths. There is an upward shift in the age groups of autopsy cases. Figure 4C illustrates the trends in the number of stillbirth cases and the national autopsy rate. The number of autopsies of stillborn infants peaked in 1988 (918 cases), and the nationwide autopsy rate of stillborn infants peaked in 1995 (1.9%).

**Figure 5:**
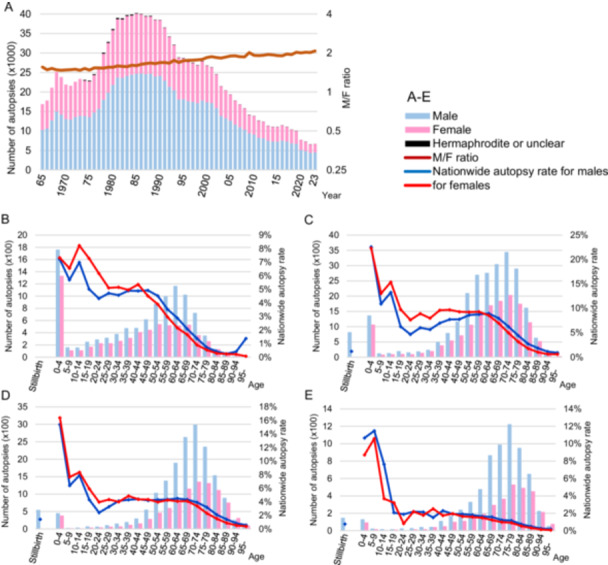
Trends in autopsies by sex and age group. (A) Trends in the number of autopsies by sex and the corresponding sex ratio. (1965–2023). Bar graphs illustrate the number of autopsies by sex, while a line graph depicts the trend in sex ratio (M/F). The sex ratio is plotted on a logarithmic scale on the vertical axis. (B–E) Number of cases and nationwide autopsy rates by age group, approximately every 20 years: (B) 1965, (C) 1980, (D) 2000, and (E) 2019. In 1965, stillbirths are included among the 0–4 age group. Stillbirths were counted regardless of sex.

When comparing autopsy numbers between sexes, the male to female ratio has gradually increased over time (1.5 in the 1960s, 2.0 in the 2020s) (Figure 5A). We examined age‐ and sex‐specific autopsy numbers (Figure 5B–E) and found that nationwide autopsy rates were higher in younger age groups. Until around 1995, autopsy rates for females were slightly higher than those for males in younger age groups. On the other hand, autopsy rates for males were slightly higher than those for females in middle to older age groups.

## 4 Discussion

### 4.1 Changes in Autopsy Numbers Before the 1980s

Since the inception of APAC‐J in 1958, there was a steady increase in registration numbers until 1968. In 1968, the Japanese Society of Internal Medicine (JSIM) launched its specialist certification system, which stipulated autopsy practice as a prerequisite for educational facility accreditation, subsequently driving a sharp rise in autopsy numbers at community hospitals [9].

Meanwhile, the university conflicts of 1968 triggered disputes within medical schools. By juxtaposing the monthly number of autopsies conducted at the University of Tokyo, where the university conflicts were particularly prominent, with the events of the medical school disputes, it becomes evident that the conflicts had a significant impact on the actual practices.

In January 1968, medical students initiated an indefinite strike, demanding improvements in their training programs [8]. In July, some students occupied Yasuda Auditorium, followed by the student occupation of the School of Medicine's main building in August. In September, the entire university was barricaded. In October, the campus‐wide student union went on strike, and the blockade continued until January 1969. The decline in the number of autopsies at The University of Tokyo in the spring and after the summer of 1968 reflects these conflicts.

At Nagoya University, the intrahospital autopsy rate had declined for several years (1968–75). This decline might be attributed to a reported delay in medical school professor appointments in Nagoya University [10], resulting in persistent staffing shortages in clinical and pathology services, which in turn led to reduced autopsy activity.

Subsequently, the internship system was abolished in 1969, and the postgraduate clinical training system commenced, significantly altering the hospital labor environment [11]. Furthermore, coinciding with this era, the implementation of the Total Staff Quota Law and the impact of the Nixon Shock led to persistent budget zero ceilings, causing the occupancy rate at The University of Tokyo Hospital to plummet to 68% [12]. These changes might affect autopsies, leading to a decline in the intrahospital autopsy rate, particularly noticeable in university hospitals. During this period, the autopsy rate in community hospitals also showed a similar decline to that in university hospitals, which is likely attributable to the significant involvement of university hospitals in dispatching physicians to community hospitals.

Nevertheless, nationwide autopsy numbers exhibited an upward trajectory until the 1980s. The introduction of the certified pathologist system by JSP in 1978 [9] played a pivotal role in significantly augmenting the volume of autopsies. The late 1970s and 1980s saw a shift, with community hospitals performing more autopsies than university hospitals, indicating a broader adoption of autopsies. Simultaneously, a clear decline in the ratio of external cases at university hospitals is apparent.

### 4.2 Since the 1980s

Since the late 1980s, the number of autopsies has been decreasing. This trend closely mirrors changes observed in other countries [1, 13]. The decline in‐hospital autopsy rates began in the 1960s, but was compensated for by an increase in the number of autopsy‐performing hospitals until the 1980s. Simultaneously, the proportion of external cases at university hospitals has declined, reflecting a broader implementation of autopsies across a wider range of hospitals. A decline in hospital autopsy rates has been reported in other countries as well, even before 1960 [14, 15, 16]. This study did not fully elucidate the causes of the long‐term decline in autopsy rates. However, we believe that many of the reasons cited in previous studies [1, 16, 17, 18] are also applicable to Japan. For example, public, clinician, and pathologist perspectives, a shift in care for older or seriously ill patients from the hospital towards long‐term facilities, financial constraints, and progress in minimally invasive diagnostic procedures [17] are all considered to be valid reasons for the decline in autopsies in Japan.

While the initial clinical training system introduced in 2004 required the completion of CPC reports and case presentations as mandatory components [19], it did not elicit a substantial increase in autopsy rates.

Despite the decrease of autopsies, the number of registering facilities increased until 2019. In Japan, the implementation status of autopsy is considered in the certification of specialists and teaching hospitals [20]. Autopsies are being performed in more hospitals, contributing to the education of more doctors and medical staff, albeit with fewer autopsies per hospital. During this period, as autopsy rates declined, the certification criteria for teaching hospitals by JSIM were gradually eased [21].

In the 2020 s, the COVID‐19 pandemic precipitated a further reduction in the autopsy rate. Despite the potential for autopsies to elucidate aspects of COVID‐19, a prevailing cautiousness regarding their execution was observed [22]. Additionally, limitations on patient admissions resulted in diminished ward occupancy. While a slight increase in autopsy numbers was observed in 2023 post‐COVID‐19 pandemic (6,557 in 2022, 6,675 in 2023), the intrahospital autopsy rate continued to decline (1.86% in 2022, 1.84% in 2023). The observed increase in autopsy numbers is plausibly associated with the recovery of ward occupancy. The late 2010s marked the commencement of deliberations regarding physician work style reforms in Japan [23]. The subsequent effects of the work style reforms on autopsy practices warrant close observation.

Japan exhibits a seasonal pattern of increased mortality during winter months, coupled with a slight reduction in autopsy rates. Consequently, the monthly autopsy count has maintained a relatively consistent distribution. The execution of autopsies necessitates labor input from both clinical and pathological personnel, suggesting a potential for unconscious modulation of workload.

Maintaining the number of autopsies requires that clinicians have a clear understanding of their significance. This study revealed that the practice of autopsies is influenced by social factors and working conditions. Both a secure society and clinicians' motivation appear to be important for sustaining autopsy practices. Pathologists can contribute by actively promoting the significance of autopsy to both clinicians and society. It is essential to carry out autopsies consistently and to ensure proper reporting of the findings. Securing allies among clinicians within hospitals or through professional societies may also facilitate greater understanding and support for autopsy practice.

### 4.3 Characteristics of Cases

While the number of autopsies and the autopsy rate have been decreasing, the proportion of brain dissections and complete torso autopsy increased since at least 2000. Autopsy is essential to confirm the precise diagnosis of brain diseases in many cases. A similar emphasis on thorough observation and elucidation of individual cases may account for the fluctuations in these proportions. The higher proportions of brain dissections in young individuals and the very elderly likely reflect, respectively, the demand for complete autopsies and the increasing prevalence of neurodegenerative diseases. However, the relatively high proportion of limited autopsies among younger age groups suggests that obtaining consent for full‐body autopsy can be more difficult in some cases. The recent rise in brain dissection rates may reflect a growing prevalence of neurological disorders due to the aging of autopsy cases. To ascertain disease prevalence, additional database investigations are required.

The actual status of ‘necropsy’ in Japan is not captured in the APAC‐J data; however, necropsies are not actively performed in Japan. Necropsy is not well suited for cases required by board‐certified pathologists, clinicians, or residents [24, 25]. Whole‐body autopsy is still considered the standard, and this likely explains the predominance of full autopsies.

Japanese life expectancy is among the highest in the world and is gradually increasing [26]. The average age of autopsy cases has also been increasing, gradually approaching the life expectancy. The age distribution of autopsy cases has changed significantly. In Japan, the period from 1971 to 1974 is known as the second baby boom. Subsequently, although there has been a gradual decline to the present, the pediatric autopsy rate remains relatively high. Even today, the autopsy rate for infants and young children remains high. There might be a strong demand for information regarding children and subsequent pregnancies.

The average age of autopsy cases is more than 10 years lower than the life expectancy. This indicates that autopsies are more frequently performed on individuals who died earlier than what is considered a full natural lifespan. Age‐stratified national autopsy rates revealed a marked elevation in younger populations. This is consistent with reports from other countries [17, 27, 28]. It is suggested that the inclination to conduct autopsies is heightened in cases of death occurring below the expected lifespan, reflecting a shared pursuit of further elucidation by clinicians and family members.

There has been a significant predominance of males in autopsy cases, with the ratio gradually increasing to 2.1 times in 2023. In the middle to older age groups, the autopsy rate among males was slightly higher than among females. Other studies also reported a higher number of autopsies conducted on males [13, 17, 27, 28, 29, 30], and a Dutch study that reported that age‐specific autopsy rates were higher among males in middle‐aged and older age groups [17]. The main reasons for the higher number of male autopsies are that autopsies are often performed on cases below the life expectancy, and more males than females die in these age groups.

The nationwide autopsy rate for female is significantly higher in younger age groups, while the autopsy rate for male is higher in middle‐aged to elderly populations. It is presumed that in younger age groups, the bereaved are often the parents of the deceased, whereas in the elderly, they are more likely to be spouses or children. There may be specific circumstances within the relationship between clinicians, patients, and families that facilitate the proposal of an autopsy.

## 5 Limitations

APAC‐J, a globally unique database, compiles autopsy data from across Japan. Although Japanese pathologists register autopsy cases voluntarily, the database includes nearly all cases owing to registration reminders and institutional accreditation requirements. While details of individual cases are limited, the database provides a broad view of autopsy practices in Japan over time.

## 6 Conclusion

This study has examined the evolution of autopsy practices in Japan over the past two‐thirds of a century. We have demonstrated that the implementation of autopsies has been influenced by various social factors, including the specialist certification system, disputes within medical schools, and the COVID‐19 pandemic. Furthermore, the age of autopsy cases has increased in line with demographic changes, and the sex distribution has also gradually shifted. In recent years, autopsies in Japan have played an increasingly important role not only in traditional contexts but also in areas such as medical accident investigations. Despite declining autopsy rates, autopsies remain essential for elucidating individual cases, advancing medical education, and addressing evolving societal and clinical needs. A peaceful society and physicians' motivation are expected to support the sustained practice of autopsies in the future.

## Author Contributions

Hiroshi Uozaki conceived and designed the study, performed the data analysis, and drafted the manuscript. Yoshinao Kikuchi and Masato Watanabe verified the underlying data and analysis. All authors contributed to the interpretation of the results and critical revision of the manuscript and approved the final version of the manuscript.

## Ethics Statement

Institutional review board approval, ethics committee approval, and informed consent were not required because the data were obtained from existing literature and publicly available data.

## Conflicts of Interest

The authors declare no conflicts of interest.

## Supporting information

PIN1‐supl.

## Data Availability

All data used in this study are publicly available as books, *Annual of the Pathological Autopsy Cases in Japan*, by the Japanese Society of Pathology (JSP). Some of the data is publicly accessible at the website of JSP in Japanese (https://pathology.or.jp/kankoubutu/autopsy-index.html) (accessed on 1 June, 2025).
