# Supplementary material for: Trends in Pathological Autopsy in Japan From 1958 to 2023
Source: Pathol Int. 2025 Jul 16;75(10):523–31. doi: 10.1111/pin.70038 (PMC12558673; doi:10.1111/pin.70038)
Supplement: Supplementary file 1 — PIN1‐supl. [file PIN-75-523-s001.pdf]

Supplementary File

(Uozaki H, Kikuchi Y, Watanabe M. Trends in Pathological Autopsy in Japan from 1958 to 2023)

Table of Contents

Figure S1: Monthly autopsy numbers (1965–2019) ·· Page 2

Figure S1: Monthly autopsy numbers (1965–2019)

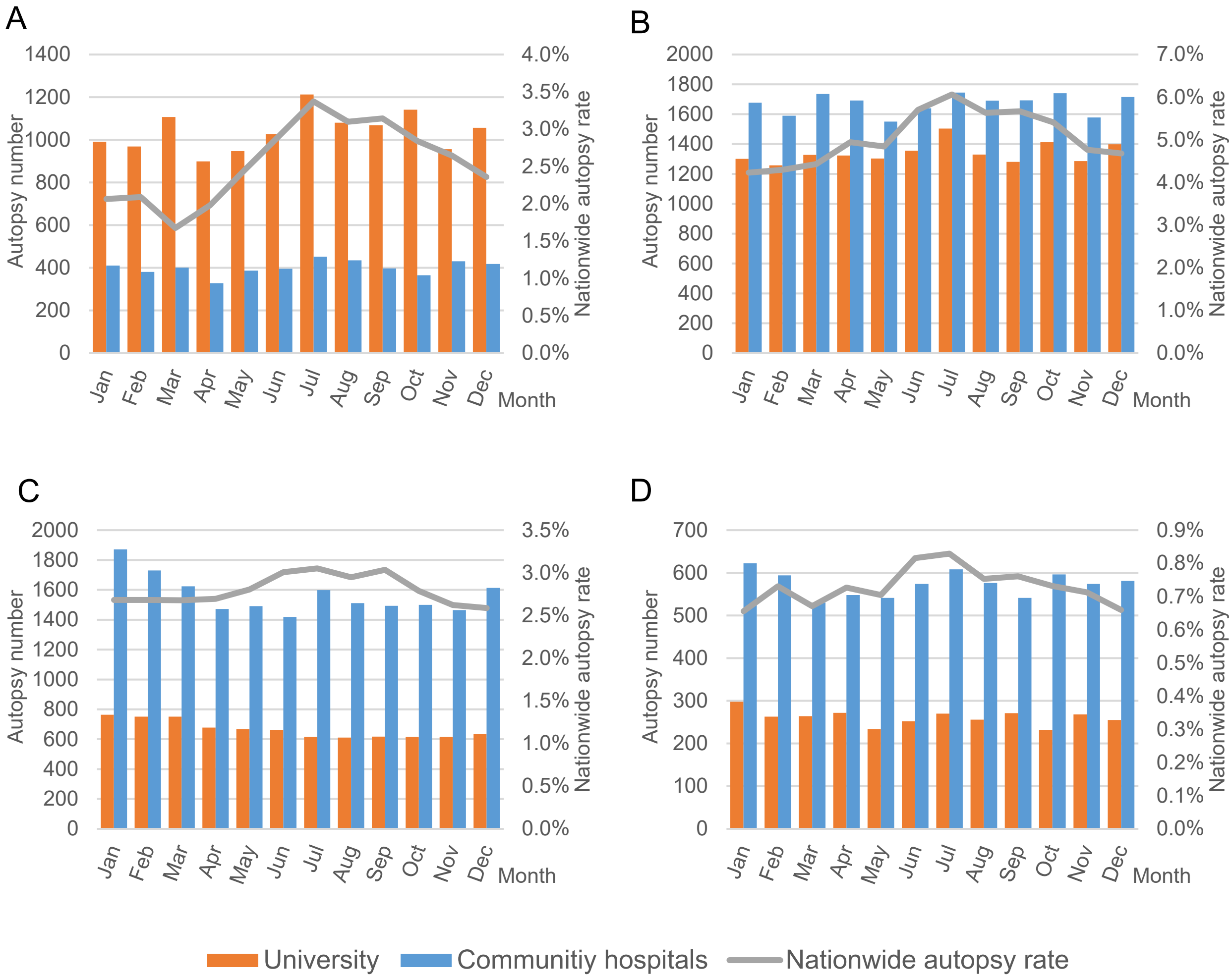

A) 1965, B) 1980, C) 2000, and D) 2019.
